# Virus-Like Nanoparticle Vaccine Confers Protection against *Toxoplasma gondii*

**DOI:** 10.1371/journal.pone.0161231

**Published:** 2016-08-22

**Authors:** Dong Hun Lee, Su Hwa Lee, Ah Ra Kim, Fu Shi Quan

**Affiliations:** 1 Department of Biomedical Science, Graduate School, Kyung Hee University, Seoul, Korea; 2 Department of Medical Zoology, Kyung Hee University School of Medicine, Seoul, Korea; Monash University, Australia, AUSTRALIA

## Abstract

The inner membrane complex (IMC) of *Toxoplasma gondii* as a peripheral membrane system has unique and critical roles in parasite replication, motility and invasion. Disruption of IMC sub-compartment protein produces a severe defect in *T*. *gondii* endodyogeny, the form of internal cell budding. In this study, we generated *T*. *gondii* virus-like particle particles (VLPs) containing proteins derived from IMC, and investigated their efficacy as a vaccine in mice. VLP vaccination induced *Toxoplasma gondii*-specific total IgG, IgG1 and IgG2a antibody responses in the sera and IgA antibody responses in the feces. Upon challenge infection with a lethal dose of *T*. *gondii* (ME49), all vaccinated mice survived, whereas all naïve control mice died. Vaccinated mice showed significantly reduced cyst load and cyst size in the brain. VLP vaccination also induced IgA and IgG antibody responses in feces and intestines, and antibody-secreting plasma cells, mixed Th1/Th2 cytokines and CD4^+^/CD8^+^ T cells from spleen. Taken together, these results indicate that non-replicating VLPs containing inner membrane complex of *T*. *gondii* represent a promising strategy for the development of a safe and effective vaccine to control the spread of *Toxoplasma gondii* infection.

## Introduction

*Toxoplasma gondii* is an obligate intracellular parasite that has adapted to infect many animal species including humans, and is capable of causing a wide spectrum of diseases, permanently infecting nearly 20% of the global population [1]. Human infection occurs through two main routes–ingestion of undercooked meat containing cysts of the parasite and ingestion of oocysts passed into the environment by cats [2]. In humans, the symptoms could be asymptomatic, resulting in a latent infection with tissue cysts. However, the infection could be severe in specific groups of patients, such individuals who are immunologically impaired due to Acquired Immunodeficiency Syndrome or organ transplants, or in congenitally infected fetuses and newborns [3,4].

Currently, the strategy of toxoplasmosis control is chemotherapy targeting the acute phase of the infection. However, the drug application has produced toxic side effects and caused re-infection [5–7]. Thus, an alternative control strategy for toxoplasmosis is urgently needed. Recent important progress has been made identifying anti-toxoplasma vaccine candidates that can stimulate an immunological response [7]. However, vaccine efficacy is not successful.

*T*. *gondii* inner membrane complex (IMC) lines the interior of the plasma membrane and contains proteins important for gliding motility and replication. Of these proteins, the IMC sub-compartment proteins (ISPs) play a role in asexual *T*. *gondii* daughter cell formation. Three proteins, IMC sub-compartment protein (ISP) 1, ISP2, and ISP3, were initially identified and shown to localize to distinct sub-compartments of the IMC in *T*. *gondii* [8]. ISP1 localizes to the apical cap portion of the IMC, ISP2 localizes to a central IMC region and ISP3 localizes to a central plus basal region of the complex. Disruption of TgISP2 markedly reduces parasite fitness and produces a severe defect in endodyogeny, the form of internal cell budding in which two daughter cells are formed within the intact mother parasite [8]. Since the amino acid sequences of ISP1, 2 and 3 are largely conserved, a vaccine targeting the three ISPs that elicits humoral or cellular immunity could have a significant impact.

Recombinant vaccines based on virus-like particles (VLPs) or nanoparticles have displayed promising safety and efficacy in preclinical and clinical studies. VLPs contain repetitive high density displays of viral surface proteins, which present conformational viral epitopes that can elicit strong T cell and B cell immune responses. In this study, for the first time, we developed VLPs containing *T*. *gondii* IMC ISP3 with influenza matrix protein 1 (M1) as a core protein. Intranasal immunization of mice with VLPs induced systemic and mucosal immune responses, including both humoral and cellular immune components. We observed that immune responses induced by the VLPs conferred protection against *T*. *gondii* (ME49) challenge infection.

## Materials and Methods

### Parasites, cells and antibodies

*Toxoplasma gondii* RH and ME49 strains were kindly provided by Dr. Ho-Woo Nam (The Catholic University of Korea, Seoul, Korea). Strains were maintained by serial intraperitoneal passage (RH) or oral passage (ME49) in Balb/C mice. *Spodoptera frugiperda* Sf9 cells were maintained in suspension in serum-free SF900 II medium (GIBCO-BRL) at 27°C in spinner flasks at 70 to 80 rpm. Horseradish peroxidase (HRP)-conjugated goat anti-mouse immunoglobulin A (IgA) and G (IgG), IgG1, and IgG2a were purchased from Southern Biotech (Birmingham, AL, USA).

### *Toxoplasma gondii* antigen

*T*. *gondii* RH tachyzoites were harvested from the peritoneal cavity of the mice 4 days after infection by injecting 1 ml of 0.1 M phosphate buffered saline (PBS, pH 7.2) as described [9]. The exudate was separated by low speed centrifugation (100×g for 5 min) at 4°C to remove cellular debris. The parasites in the supernatant were precipitated by centrifugation at 600×g for 10 min, and washed in PBS and sonicated.

### Cloning of *Toxoplasma gondii* IMC and influenza M1 genes

*Toxoplasma gondii* RH tachyzoites were collected from mice and total RNA of tachyzoites was extracted (RNeasy Mini kit; Qiagen, Valencia, CA, USA). Complementary DNA (cDNA) was synthesized using a Prime Script 1^st^ Strain CDNA Synthesis Kit (Takara, Japan). *Toxoplasma gondii* IMC gene was amplified by polymerase chain reaction (PCR) from cDNA with primers 5-AAAGAATTCACCATGGGGAACACGGCGTGCTG-3 (EcoRI and XhoI underlined) and 5-TTACTCGAGTTAGTTTCTGTCGTTGCTTGC-3 (EcoRI and XhoI underlined). A cDNA fragment containing the gene was cloned into pFastBac vector (Invitrogen, Carlsbad, CA, USA) as described previously [10]. For influenza M1 gene cloning, A/PR/8/34 virus was inoculated into MDCK cells and total viral RNA was extracted as mentioned above. A cDNA fragment containing the M1 was cloned into pFastBac vector (Invitrogen, Carlsbad, CA, USA).

### Generation of recombinant baculovirus (rBV)

rBVs expressing *T*. *gondii* IMC or influenza M1 were generated as described previously [11]. Transfection of DNA containing these genes was done using Cellfectin II (Invitrogen) with SF9 cells as recommended by the manufacturer, followed by transformation of pFastBac containing *T*. *gondii* IMC or M1 with white/blue screening. The rBVs were derived using a Bac-to-Bac expression system (Invitrogen) according to the manufacturer’s instructions.

### Production of VLPs

VLPs were produced in Sf9 insect cells co-infected with recombinant rBVs expressing *T*. *gondii* IMC and M1. VLPs released into the cell culture supernatants were harvested and purified through a 15%-30%-60% discontinuous sucrose gradient at 28000 rpm for 1 h at 4°C. VLP bands between 30% and 60% were collected and then diluted with PBS and pelleted at 28000 rpm for 1 h at 4°C. VLPs were resuspended in PBS overnight at 4°C.

### Characterization of VLPs

VLPs were characterized by Western blots and electron microscopy. For Western blot analysis, antibody was collected prepared from *T*. *gondii* ME 49 infected mice. The antibody was used to probe for *T*. *gondii* IMC protein. Anti-M1 antibody was used to determine M1 protein content. Negative staining of VLPs was performed followed by transmission electron microscopy, which was done at the Korea Advanced Institute of Science and Technology.

### Immunization and Challenge

Female, 6–8 week old BALB/c mice (NARA Biotech, Seoul, Korea) were used. Groups of mice (n = 6 per group) were intranasally immunized twice with 100 μg total VLP protein at 4-week intervals. Blood samples were collected by retro-orbital plexus puncture before immunization and at 1, 2 and 4 weeks after priming and boosting. For challenge studies, naive or vaccinated mice were infected with *T*. *gondii* ME49 orally with 30 or 120 cysts in 100 μl PBS. Mice were observed daily to record body weight changes and survival. We followed an approved Kyung Hee University IACUC protocal for this study.

### Sample collection, antibody responses in sera, feces and intestines

Blood and feces samples were collected at weeks 1, 2 and 4 after immunization and at weeks 1 and 4 after challenge infection. Intestine samples were collected at week 4 postchallenge. Feces samples were incubated in PBS at 37°C for 1 h and the supernatants were collected after centrifugation at 2000 rpm and stored -20°C until use. The small intestine site for each mouse was 10 cm beneath the stomach. The collected intestine was incubated in 5 ml PBS at 37°C for 1 h, and the supernatants were collected at 2000 rpm for 10 min. IgG, IgG1, IgG2a and IgA antibody responses were determined by ELISA. Briefly, 96-well microtiter plates were coated with 100 μl of soluble tachyzoite at a concentration of 2 μg/ml in coating buffer at 4°C degree overnight. The samples were diluted to 100x for sera, and 10x for intestines and feces, and added onto plates. The plates were then incubated with HRP-conjugated secondary antibodies as described previously [12].

### Analysis of antibody-secreting cell response in vitro

To detect antibody-producing cells (ASC), diluent or *T*. *gondii* RH (2 μg/ml in 100 μl) was used to coat 96-well culture plates. Freshly isolated cells from the spleen (1 × 10^6^ cells/well) were added to each well and incubated for 3–4 days at 37°C with 5% CO_2_. Parasite-specific antibody secreted into the culture medium and bound to the coated antigens were determined. Horseradish peroxidase-conjugated anti-mouse immunoglobulin antibodies were used to determine ASC responses.

### T cell responses

Individual mouse spleen was collected 1 month after challenge from the immunized and naïve groups. Single-cell suspensions were prepared from each spleen. Cells were incubated in 96-well flat culture plates for 3–4 days at 37°C in the presence of 5% CO_2_. Cells in 100 μl of RPMI-1640 were stimulated with 100 μl of 0 or 2 μg/ml *T*. *gondii* RH. For the cytokine assay, supernatants of spleen cell cultures were collected from each well by separation and stored at -20°C until use. OptEIA sets (BD Bioscience, San Jose, CA, USA) were used to determine the concentration of interferon-gamma (IFN-γ), interleukin (IL)-6 and IL-10 in culture supernatants following the manufacturer’s procedures. To determine CD4+ and CD8+ T cells, single-cell suspension from spleen were stained with CD8 (BD Biosciences) and analyzed using a FACScan flow cytometer (BD, Mountain View, CA, USA). Results were analyzed using WinMDI 2.9 software (De Novo Software, Los Angeles, CA, USA).

### Statistics

All parameters were recorded for individuals within all groups. Statistical comparisons of data were carried out using the One-way ANOVA (Tukey) or student t-test of PC-SAS 9.3. A P value < 0.05 was considered to be significant.

## Results

### Generation of recombinant constructs

**Fig 1:**
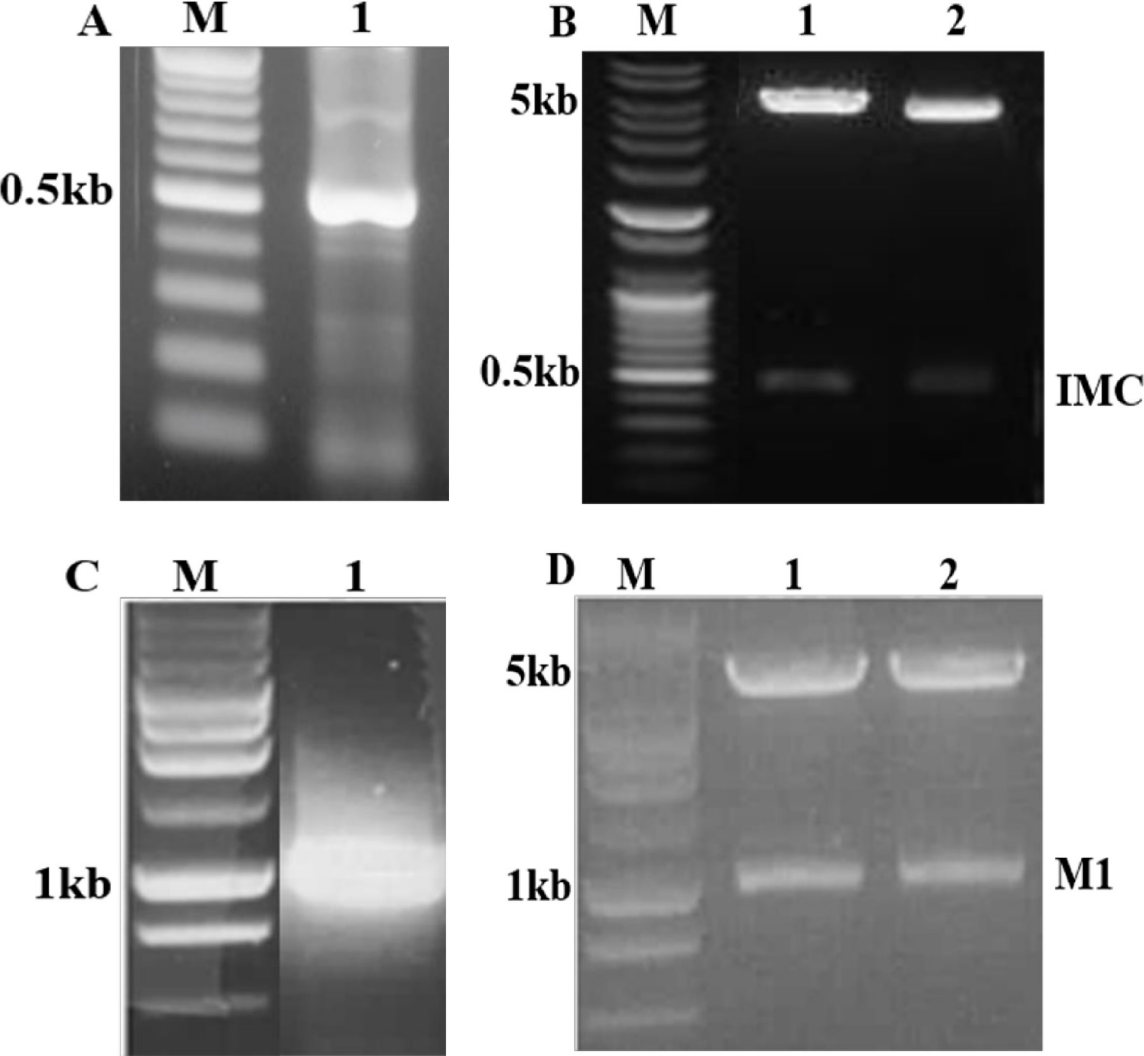
Construction of pFastBac vectors. (A) *Toxoplasma gondii* IMC gene was PCR-amplified from cDNA synthesized using a Prime Script 1st Strain CDNA Synthesis Kit using total RNA extracted from *T*. *gondii* RH. M denotes DNA marker and lane 1 indicates amplified PCR product. (B) Influenza M1 gene was PCR amplified from total RNA extracted from influenza virus (A/PR/8/34). M denotes DNA marker and lane 2 indicates amplified PCR product. (C and D) *Toxoplasma gondii* IMC gene and influenza M1 gene were cloned in to pFastBac with EcoRI / XhoI and EcoRI / XhoI enzymes, respectively, resulting in *T*. *gondii* IMC plasmid (C) and M1 plasmid (D).

*Toxoplasma gondii* IMC and influenza M1 genes were amplified by PCR (Fig 1A and 1C) and cloned into pFastBac vectors (Fig 1B and 1D). The recombinant plasmids were digested with EcoR I and Xho I and the results revealed that the recombinant plasmid contained inserts (Fig 1B and 1D). Sequence analysis indicated that the nucleotide sequences of the *T*. *gondii* IMC and influenza M1 genes were identical to previously published sequences (accession numbers HQ012579 for *T*. *gondii* IMC and EF467824 for M1).

### Characterization of *T*. *gondii* VLPs

**Fig 2:**
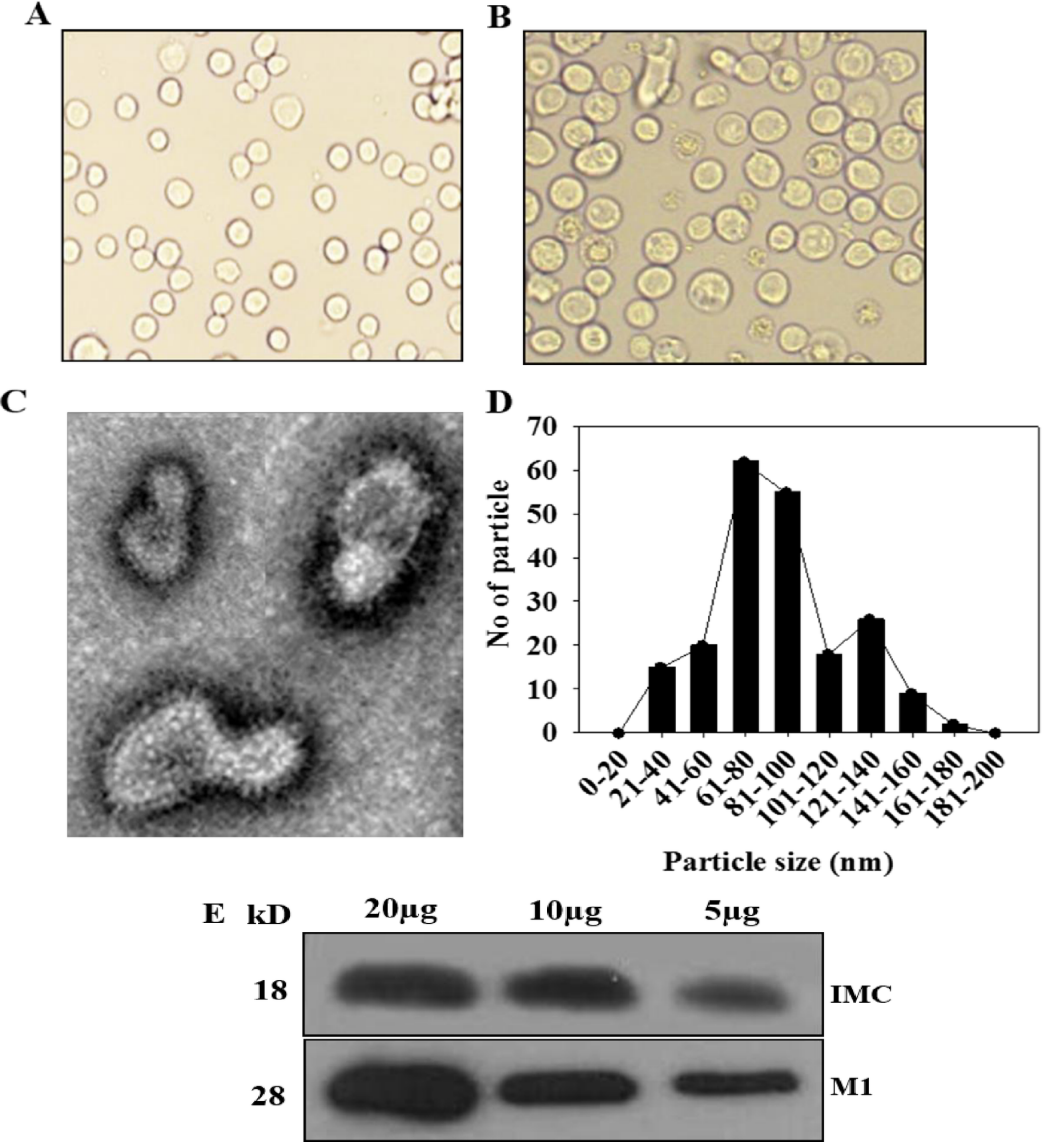
Characterization of virus-like particles (VLPs). (A) Normal SF9 cells. (B) VLP-producing cells. (C) Electron microscopy and (D) VLP size determination. Negative staining of VLPs was performed followed by transmission electron microscopy. The size is between 40 and 120 nm. (E) Western blot analysis. VLPs (20, 10, 5 μg) were loaded for SDS-PAGE. Polyclonal mouse anti*-T*. *gondii* antibody was used to probe *T*. *gondii* IMC protein (18kD) and anti-M1 monoclonal antibody was used to determine influenza M1 protein (28kD).

We produced VLPs in insect cells co-infected with recombinant baculoviruses expressing *T*. *gondii* IMC and influenza M1. VLP-producing Sf9 cells (Fig 2B) were much bigger than normal control cells (Fig 2A). *T*. *gondii* IMC VLPs showed spherical shapes with spikes on their surfaces under electron microscopy (Fig 2C). VLPs generated resemble virions in morphology and size. The incorporation of *T*. *gondii* IMC and M1 into VLPs was confirmed by Western blot using anti *T*. *gondii* polyclonal antibody and M1 monoclonal antibody (Fig 2D).

### Immunization with VLPs elicits antibody responses

**Fig 3:**
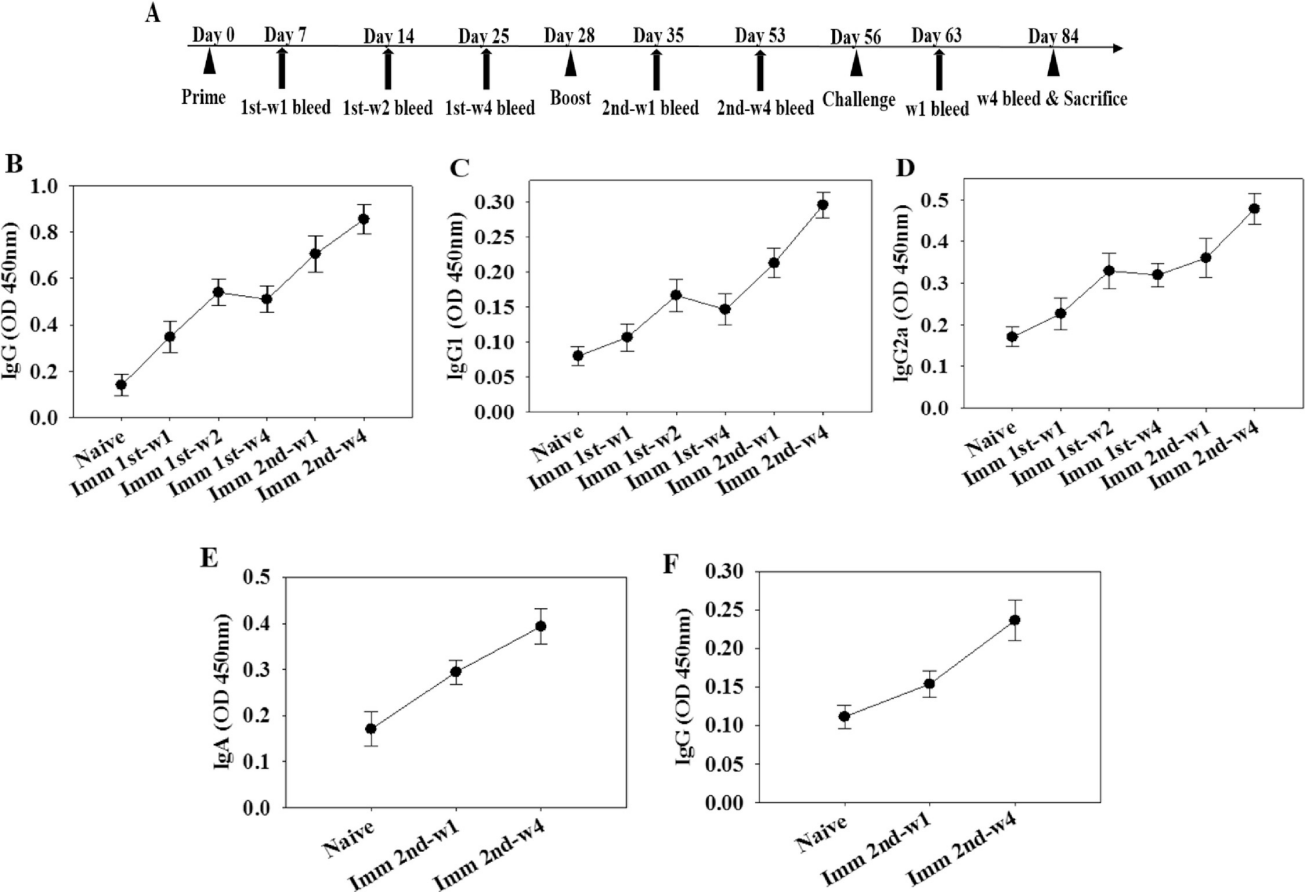
*Toxoplasma gondii*-specific antibodies responses upon immunization. Mice were immunized twice with VLPs as indicated with a 4-week interval. *T*. *gondii*-specific IgG, IgG1 and IgG2a antibody responses in the sera were determined after prime and boost (A-C). *T*. *gondii*-specific IgG and IgA antibody responses in the feces were determined (D and E). Imm: immunization

The experimental schedule and the levels of *T*. *gondii*-specific IgG, IgG1, IgG2a in sera and IgA and IgG antibody responses in feces after priming and boosting were determined as shown in Fig 3. Total IgG and IgG1, and IgG2a responses from the mice immunized with VLPs showed significantly higher titers after boost compared with those after prime at week 1 and 4, indicating the progressive maturation of *T*. *gondii* IMC-specific antibody (Fig 3B–3D). Also, higher levels of IgA and IgG responses in feces were observed after boost compared to naïve control (Fig 3E and 3F), indicating mucosal immunity was elicited. These results indicate that VLPs containing *T*. *gondii* IMC are highly immunogenic against *T*. *gondii*, resulting in higher levels of systemic and mucosal antibody responses.

### High levels of *T*. *gondii*-specific antibodies are induced upon challenge infection

**Fig 4:**
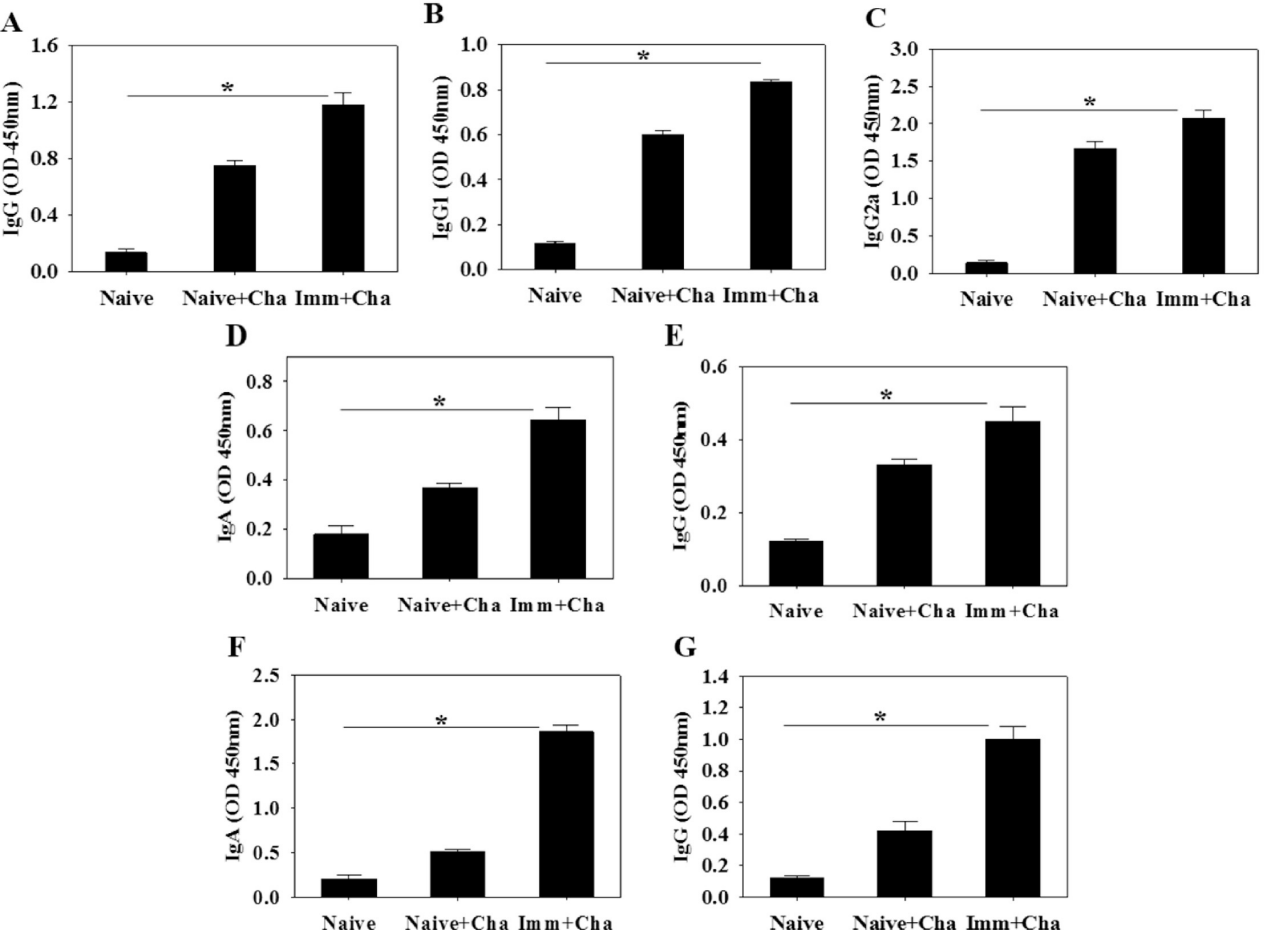
*Toxoplasma gondii*-specific antibodies responses upon challenge infection. Immunized mice were challenge infected orally with *T*. *gondii* ME49 at week 4 after boost and *T*. *gondii*-specific IgG (A), IgG1 (B) and IgG2a (C) antibody responses in the sera were determined (A-C; mean±SD, *P < 0.05). IgA and IgG antibody responses from feces and intestine were also determined at week 4 postchallenge (D-G; mean±SD, *P < 0.05). Cha: challenge infection

To determine antibody response profiles in serum, feces and intestines upon challenge infections, groups of mice were orally challenge infected with *T*. *gondii* ME49 cysts at week 4 after boost. Significantly higher levels of IgG, IgG1 and IgG2a antibody responses reactive to *T*. *gondii* antigen in sera were detected at week 4 upon challenge (Fig 4A–4C; *P < 0.05). Since intranasal immunization is believed to occur via the mucosal route, we measured *T*. *gondii*-specific IgA and IgG antibody responses in mucosal sites in feces and intestines. Higher levels of *T*. *gondii*-specific IgA and IgG antibodies were detected in feces (Fig 4D and 4E; *P < 0.05) and intestines (Fig 4F and 4G; *P < 0.05). These results indicate that IgA and IgG antibodies were rapidly boosted by subsequent infection with *T*. *gondii*. Vaccinated mice displayed higher levels of systemic and mucosal antibody responses upon challenge infections.

### Immunization with VLPs confers protection from *T*. *gondii* challenge infection

**Fig 5:**
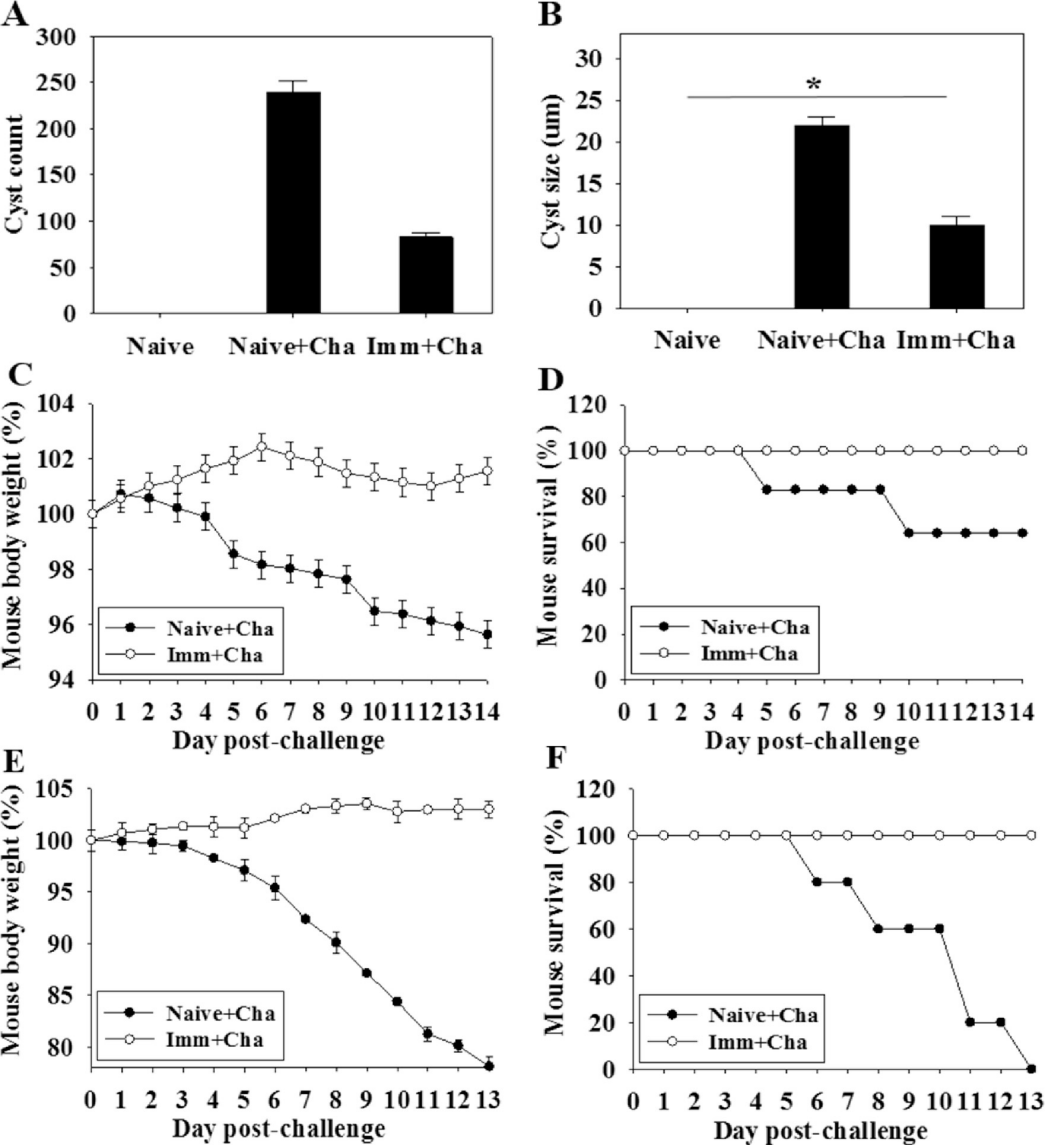
Protections against *T*. *gondii* (ME49) challenge infection. Immunized mice were challenge infected orally with *T*. *gondii* (ME49) with a lethal dose (120 cysts) or low dose (30 cysts) at week 4 after boost. The experiment was repeated twice. For a low dose challenge experiment, mice were sacrificed and cyst counts (A) and cyst sizes (B) in brain were determined at week 4 after challenge infections (mean±SD, *P < 0.05, *P < 0.05). Body weight changes and survival were also determined daily upon challenge infection (C and D). For a lethal dose challenge experiment, mice were monitored daily to determine body weight changes and survival (E and F). Upon the lethal dose challenge infection (120 cysts), all vaccinated mice survived without significant decrease of body weight, whereas all naïve control mice died with a significant and progressive loss in body weight (E and F).

Cyst load and cyst size in the brain following challenge infection are the most important indicators to assess VLP vaccine protective efficacy. Immunized mice were orally challenge infected with *T*. *gondii* ME49 cysts at 4 weeks after boost. Cyst counts and sizes in brain samples were determined at 1 month postchallenge. As shown in Fig 5A and 5B, significantly decreased cyst counts and cyst sizes in brain were detected in mice upon challenge infection compared to non-immunized mouse controls (Reduction rate: 65%; Fig 5A and 5B, *P < 0.05), indicating that protective efficacy was induced after challenge infection. Body weight changes were also determined after challenge infections. As shown in Fig 5C, immunized mice gained body weight, whereas control mice lost body weight or died upon challenge. All mice immunized survived whereas control mice showed 60% survival (Fig 5D). Upon lethal dose challenge infection (120 cysts), all vaccinated mice survived without significant decrease of body weight, whereas all naïve control mice died with a significant and progressive loss in body weight (Fig 5E and 5F). VLP immunized mice showed significantly decreased cyst counts and cyst sizes, and all immunized mice survived upon lethal challenge infection, indicating protective immunity was induced against *Toxoplasma gondii* infection.

### Infection with *Toxoplasma gondii* induces a marked anti-*T*. *gondii* antibody-secreting cell response

**Fig 6:**
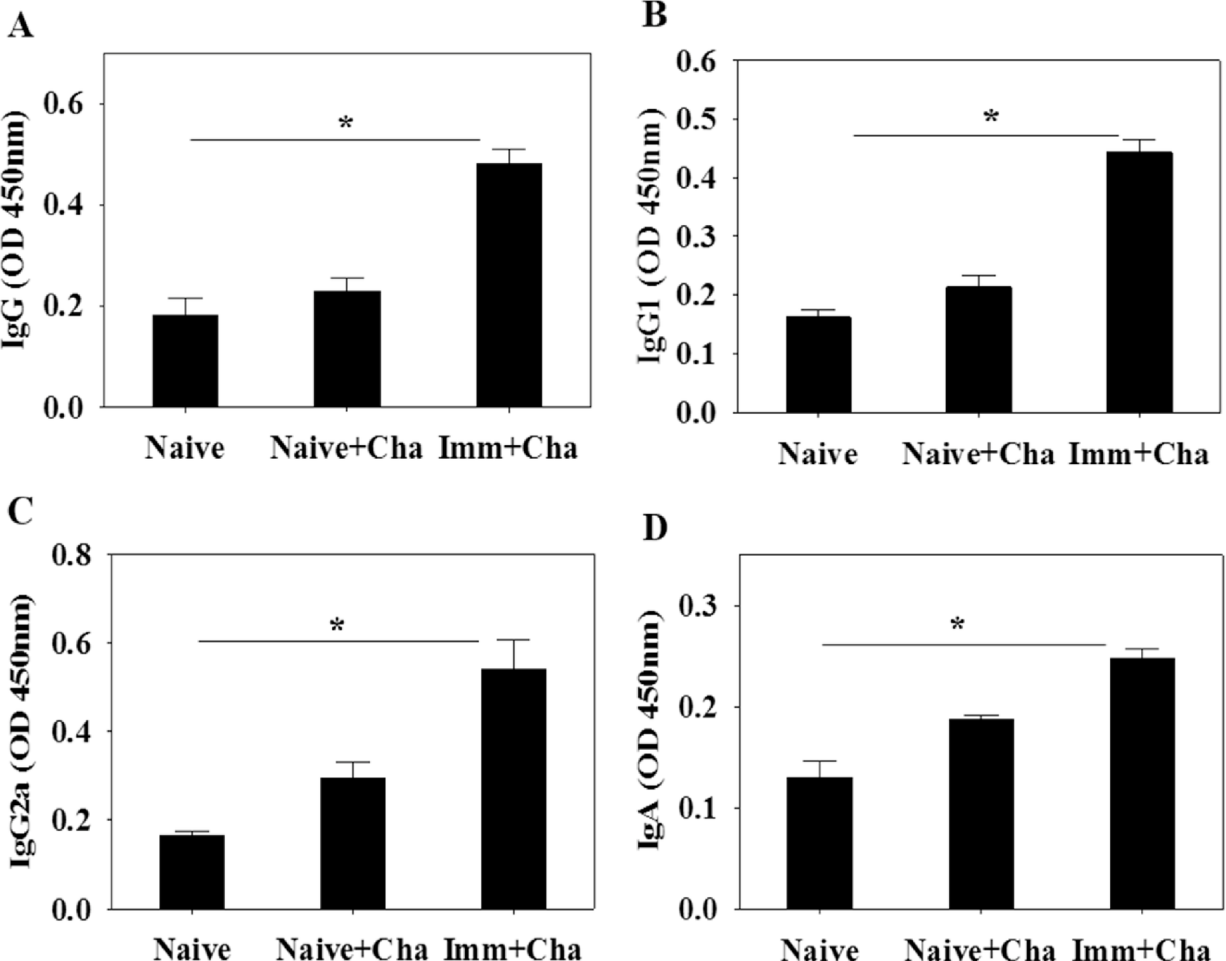
Antibody secreting cell responses. Immunized mice were challenge infected with *T*. *gondii* (ME 49) at week 4 after boost and antibody-secreting cells (ASC) in the spleen were determined at week 4 after challenge infections. IgG, IgG1, IgG2a and IgA-secreting cell responses were seen as indicated (A-D; mean±SD, *P < 0.05).

To determine antibody-secreting cell responses after challenge infection with *T*. *gondii*, spleen cells were collected from mice and subjected to in vitro culture. Significantly higher levels of IgG, IgG1 and IgG2a antibodies specific to *T*. *gondii* were secreted into culture supernatants by spleen cells of immunized mice than from cells derived from mice infected only with *T*. *gondii* (Fig 6A–6C; *P < 0.05). Significant levels of IgA antibodies specific to *T*. *gondii* were detected in the same supernatants after 4 days of culture (Fig 6D; *P < 0.05). Taken together, these results indicate that B cells have the capacity to rapidly differentiate into antibody-secreting cells upon infection with *T*. *gondii*.

### Cytokine production and T cell responses

**Fig 7:**
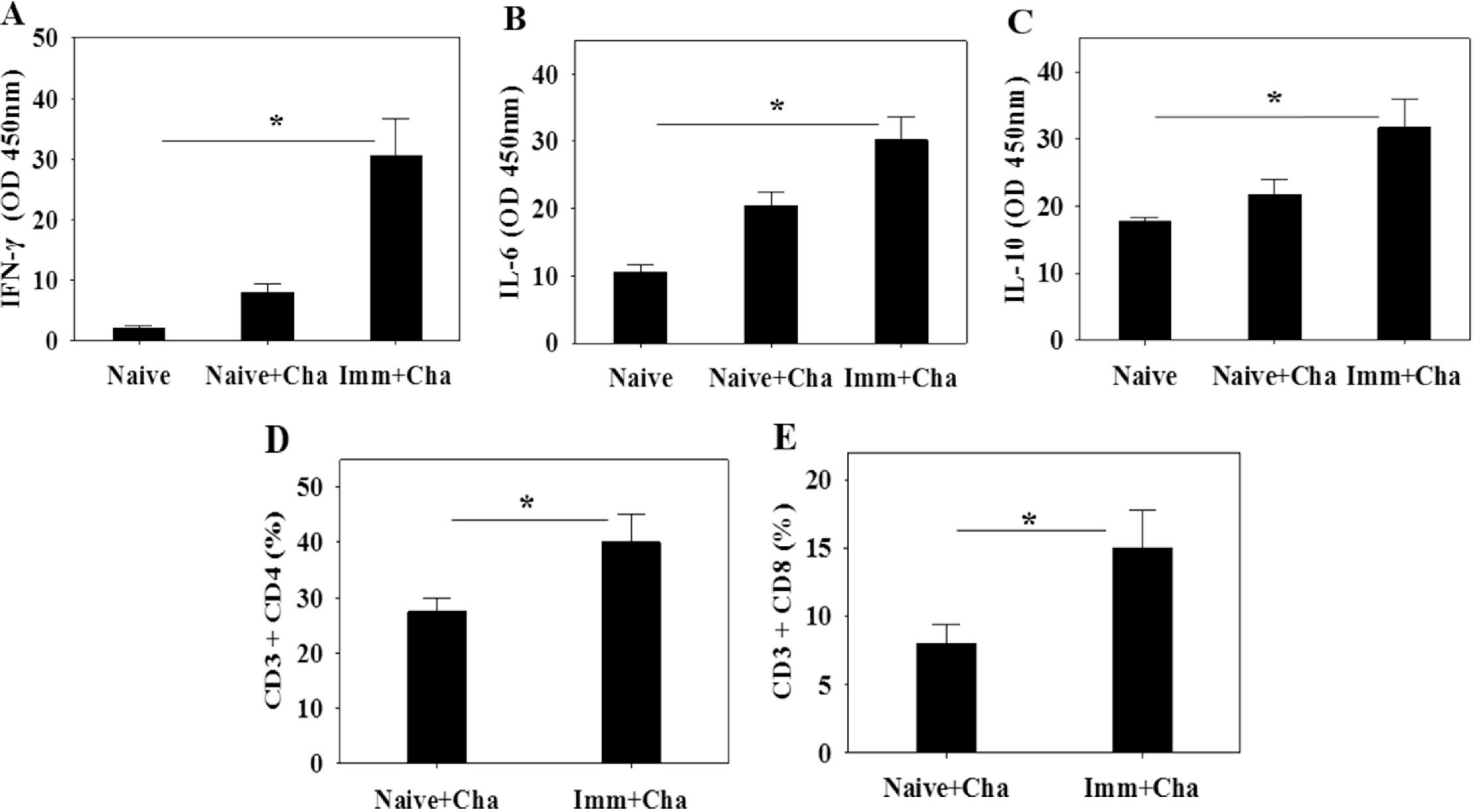
T cell responses. Immunized mice were challenge infected and at week 4 after challenge infections mice were sacrificed. IFN-γ, IL-4 and IL-6 cytokines (A-C), and CD4+ T cell and CD8+ T cell populations in the spleen were determined (D and E; mean±SD, *P<0.05).

To evaluate cytokine production, splenocytes were harvested 1 month after challenge infection. The levels of production of IFN-γ, IL-6 and IL-10 from cytokine-secreting cells were determined. As shown in Fig 7, significantly higher levels of the three cytokines were produced following *T*. *gondii* antigen stimulation compared to controls (Naïve + Cha) (Fig 7A–7C; *P < 0.05), indicating that immunized mice induced higher levels of mixed Th1/Th2 cytokine responses upon challenge infections. Both CD4+ T cells and CD8+ T cells were raised from immunized mice upon challenge infections compared to non-immunized control mice (Fig 7D and 7E; *P < 0.05).

## Discussion

Virus-like particle (VLP) vaccines can stimulate strong humoral and cellular immune responses, representing one of the most appealing approaches for a vaccine platform [13]. VLPs lack viral genome, and mimic the key structural and functional features of viruses, representing a safe and effective vaccine platform [13,14]. In addition, VLPs can be produced in insect cell expression systems, where foreign antigens can be displayed. Thus, for the first time, we generated influenza M1 VLPs consisting of foreign antigen *T*. *gondii* IMC. We found that VLPs elicited significant levels of *T*. *gondii*-specific total IgG, IgG1 and IgG2a antibody responses in the sera and IgA antibody responses in the feces. *T*. *gondii* (ME49) replication was significantly inhibited in the brain, resulting in complete protection upon challenge infection.

IMC ISPs are known to be crucial for the replication of the apicomplexan parasite *T*. *gondii* [8]. The IMC lines the interior of the plasma membrane and contains proteins important for gliding motility. Of these proteins, the IMC ISPs have recently been shown to play a role in asexual *T*. *gondii* daughter cell formation. Influenza virus matrix (M1) protein core in RSV VLPs has successfully formed spherical shapes, in which RSV VLPs significantly decreased lung virus loads upon live virus challenge [15]. Thus, we used influenza matrix M1 as a core protein to generate *T*. *gondii* VLPs where repeated structure of inner membrane complex sub-compartment proteins (IMC ISP) was supposed to be on the surface of VLPs. We assume that IMC ISP in virus-like nanoparticle form could be an important immunogen in which VLPs could induce humoral or cellular immunity, resulting in protective immunity. Encouragingly, as shown in Fig 2C, VLPs showed spherical particle shape, where repeated structure of IMC ISP was clearly seen on the surface along with M1 core protein. In the current study, intranasal immunization of VLPs induced worm reduction in the brain with 100% survival against *T*. *gondii* (ME49) challenge infection. IMC ISP proteins in VLPs presented as a repetitive, particulate virus-like structure; they might be highly immunogenic and induce protective humoral, cellular, and mucosal immune responses [16, 17]. Immunity against inner membrane complex sub-compartment proteins induced by VLP vaccination might successfully inhibit the replication of *T*. *gondii* in a mouse model.

In this study, VLP vaccination elicited significant levels of IgG2a dominant *T*. *gondii*-specific IgG antibody responses upon challenge infection (Fig 4C), which might contribute to significantly reduced cyst counts and cyst size in the brain. IgG2a-dominant response indicates Th1-type immune response. Humoral antibody responses induced by VLPs limited the multiplication of *T*. *gondii*. *T*. *gondii* can be killed by activated complement system or phagocytosis and macrophage killing [18–20]. To induce mucosal immunity, intranasal vaccination was applied. As indicated in Figs 3E and 3F and 4D–4G, *T*. *gondii*-specific IgG and IgA antibodies were detected in feces and intestine, which might also contribute to the protection. Since there is no standard protocol for the evaluation of vaccines against *T*. *gondii* in animal models, the *T*. *gondii* isolate and dose used for challenge varies between studies [21]. We used 30 and 120 *T*. *gondii* ME49 cysts for challenge infection, respectively. Vaccinated mice showed complete protection resulting in gaining weight with 100% survival, while non-vaccinated mice showed weight loss with 60% and 0% survival, respectively (Fig 5D and 5F). Chimeric recombinant VLPs containing *Plasmodium falciparum* circumsporozoite protein antigen fused to hepatitis B surface antigen has reached a Phase III clinical trial of malaria vaccine, in which 30–50% protection was elicited, which is considered to be significant [22]. By the same token, we could conclude that our *T*. *gondii* VLPs containing IMC ISP is an attractive vaccine candidate.

A good vaccine should induce memory immune responses, which can provide effective protective immunity. A fraction of memory B lymphocytes developed in the secondary lymphoid organs is routed to the spleen, resides there as long-lived plasma cells, and secretes antibodies, maintaining long-term serum antibody levels [23, 24]. We observed the presence of *T*. *gondii*-specific antibody-secreting plasma cells in the spleen of the VLP-immunized mice and found that VLP-immunized mice were protected. Taken together, the results suggest that *T*. *gondii* VLPs can induce the differentiation of B cells to long-lived plasma cells secreting antibodies, which may play a role in maintaining effective protective immunity.

To evaluate cell-mediated immunity induced by VLPs in this model, Th1-like cytokines IFN-γ and Th2-like cytokines IL-6 and IL-10 were determined when splenocytes were stimulated with *T*. *gondii* antigen. Mixed Th1/Th2-like cytokines were induced in present study (Fig 7). It is likely that activated T-helper cells activate B cells and the interaction between the T-helper cells and B-cells cause the B-cells to differentiate into plasma cells and memory cells [25]. To determine the potential role of CD4+ T and CD8+ T cells in conferring protective immunity, CD4+ and CD8+ T cells population from immunized mice were detected. Increased CD4+ T and CD8+ T cells in immunized mice may contribute to the protection against *T*. *gondii* ME49 challenge infections.

*T*. *gondii* produces a high burden of disease in some areas of the world. Development of vaccines *T*. *gondii* vaccines in humans is a high priority. Since vaccine study reported on *T*. *gondii* have not been able to induce effective protective immunity [26], developing effective vaccine against *T*. *gondii* infection would have significant impact. Our results show that *T*. *gondii* VLPs can induce systemic and mucosal humoral responses, and cellular immune responses capable of conferring protection against *T*. *gondii* ME49 challenge infection. These results provide insight for developing effective prophylactic vaccines based on VLPs to fight pathogenic *T*. *gondii*.
