# Correction: Virus-Like Nanoparticle Vaccine Confers Protection against *Toxoplasma gondii*

**DOI:** 10.1371/journal.pone.0301214

**Published:** 2024-03-21

**Authors:** Dong Hun Lee, Su Hwa Lee, Ah Ra Kim, Fu Shi Quan

In Fig 1, the images for Fig 1B and Fig 1C are incorrectly switched. The image that appears as Fig 1B should be Fig 1C, and the image that appears as Fig 1C should be Fig 1B. Please see the correct Fig 1 here.

**Fig 1:**
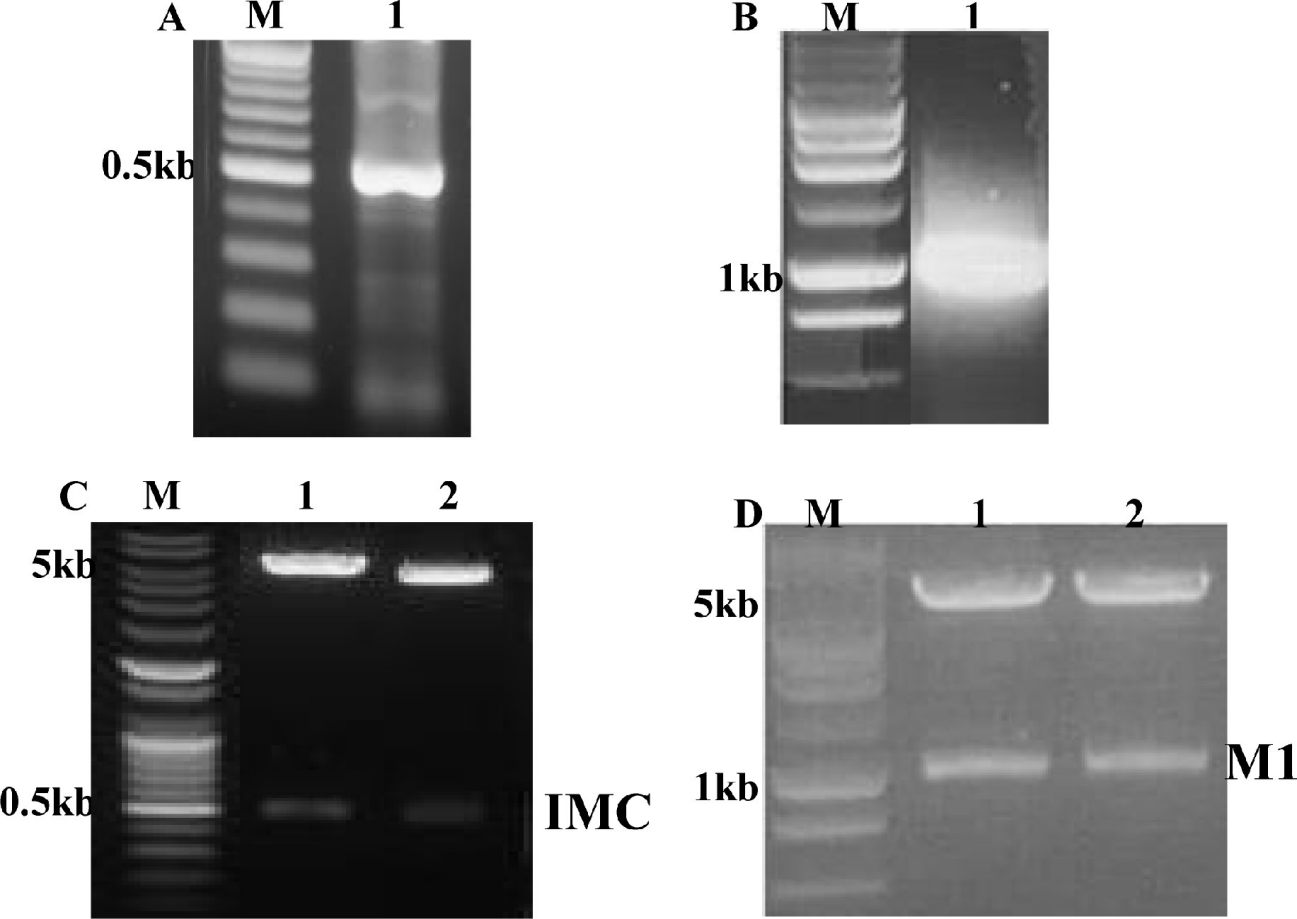
Construction of pFastBac vectors. (A) Toxoplasma gondii IMC gene was PCR-amplified from cDNA synthesized using a Prime Script 1st Strain CDNA Synthesis Kit using total RNA extracted from T. gondii RH. M denotes DNA marker and lane 1 indicates amplified PCR product. (B) Influenza M1 gene was PCR amplified from total RNA extracted from influenza virus (A/PR/8/34). M denotes DNA marker and lane 2 indicates amplified PCR product. (C and D) Toxoplasma gondii IMC gene and influenza M1 gene were cloned in to pFastBac with EcoRI / XhoI and EcoRI / XhoI enzymes, respectively, resulting in T. gondii IMC plasmid (C) and M1 plasmid (D).
